# Fourteen Draft Genome Sequences for the First Reported Cases of Azithromycin-Resistant *Neisseria gonorrhoeae* in Ireland

**DOI:** 10.1128/genomeA.00403-17

**Published:** 2017-06-08

**Authors:** Micheál Mac Aogáin, Nicholas Fennelly, Anne Walsh, Yvonne Lynagh, Michaël Bekaert, Brendan Lawlor, Paul Walsh, Brian Kelly, Thomas R. Rogers, Brendan Crowley

**Affiliations:** a Department of Clinical Microbiology, Trinity Translational Medicine Institute, School of Medicine, Trinity College Dublin, Dublin, Ireland; b Virology Laboratory, St. James’s Hospital, Dublin, Ireland; c NSilico, Nova Center, Belfield Innovation Park, Dublin, Ireland

## Abstract

Here, we report the draft genome assemblies of 14 azithromycin-resistant *Neisseria gonorrhoeae* clinical isolates, representing the first such strains identified in Ireland. Among these isolates are the first reported highly resistant strains (MIC >256 mg/liter), which both belonged to the ST1580 sequence type.

## GENOME ANNOUNCEMENT

*Neisseria gonorrhoeae* remains an urgent health care challenge in the face of emerging resistance to first-line therapeutic agents. Resistance to azithromycin (AZT), an antibiotic used in dual treatment regimes, represents a worrying development (1). We have previously described two clinical cases involving highly AZT-resistant *N. gonorrhoeae* isolates belonging to ST1580—the first such cases identified in Ireland (2, 3). These strains were identified during surveillance of drug-resistant *N. gonorrhoeae* and noted for their high-level resistance (MIC>256 mg/liter). Here, we report genome assemblies for these and 12 additional resistant strains detected in Ireland (MICs ranging from 1 to 16 mg/liter).

Sequencing libraries of *N. gonorrhoeae* genomic DNA were generated using the NexteraXT library preparation kit (Illumina, Eindhoven, the Netherlands) and sequenced on an Illumina MiSeq instrument at the TrinSeq sequencing lab (Trinity College Dublin) using MiSeq v3 reagents (300-base paired-end run). Genome sequence assembly, analysis and automated reporting were carried out using the Simplicity analysis platform (NSilico Lifescience Ltd., Ireland)—pipeline v1.4 (4). Genome assemblies are detailed in Table 1. Strain multilocus sequence types (MLST) were determined using pubMLST (http://pubmlst.org/neisseria/—database release 2017/4/10) (5). Sequencing reads were also aligned to the *N. gonorrhoeae* NCCP11945 genome (CP001050) using the Burrows-Wheeler short-read aligner (BWA, version 0.7.12-r1039) and interstrain genetic variations, including those present in antibiotic resistance genes, were resolved using SAMtools (version 0.1.19-96b5f2294a) (6, 7).

**TABLE 1:** Genomic sequence assembly overview

Strain	Yr isolated	Specimen type	AZT MIC (mg/liter)	23S rRNA mutations^a^		MLST	Assembly size (bp)	Fold coverage	% G+C	No. of contigs	*N*_50_ (bp)	Size of largest contig (bp)	GenBank accession no.
				A2059G	C2611T								
NGSJH7	2008	Urethral swab	>256	+ (3/4)	−	ST1580	2,075,384	173×	52.69	120	33,484	68,114	NAGL00000000
NGSJH11	2014	Urethral swab	>256	+	−	ST1580	2,079,908	116×	52.76	145	22,826	139,128	NAGP00000000
NGSJH13	2008	Urethral swab	16	−	−	Novel ST^b^	2,139,248	147×	52.63	131	30,939	106,477	NAGR00000000
NGSJH16	2008	Urethral swab	16	−	+	ST7822	2,120,180	166×	52.54	125	35,303	108,373	NAGT00000000
NGSJH5	2011	Urethral swab	12	−	+	ST9363	2,097,066	130×	52.75	147	23,449	75,854	NAGK00000000
NGSJH9	2014	Urethral swab	8	−	+ (2/4)	ST1901	2,120,291	147×	52.5	122	32,984	105,727	NAGN00000000
NGSJH12	2014	Urethral swab	8	−	+	ST1587	2,131,824	114×	52.62	121	26,278	82,135	NAGQ00000000
NGSJH8	2011	Urethral swab	2	−	−	ST1579	2,090,734	158×	52.69	135	23,447	105,772	NAGM00000000
NGSJH4	2012	Urethral swab	2	−	+	ST9363	2,053,541	130×	52.83	119	27,039	108,385	NAGJ00000000
NGSJH1	2009	Urethral swab	1	−	−	ST1901	2,127,937	72×	52.47	131	27,371	108,554	NAGH00000000
NGSJH2	2009	Urethral swab	1	−	−	ST1582	2,108,844	186×	52.65	105	35,856	175,104	NAGI00000000
NGSJH10	2014	Urethral swab	1	−	−	ST9363	2,075,749	161×	52.69	111	34,949	106,004	NAGO00000000
NGSJH14	2014	Urethral swab	1	−	−	ST9363	2,069,975	173×	52.73	113	35,282	139,973	NAGS00000000
NGSJH17	2014	Urethral swab	1	−	−	ST7363	2,125,521	146×	52.54	121	34,743	105,124	NAGU00000000

a +, supported by 100% of mutant calls; (3/4), supported by 75% of mutant calls; (2/4), supported by 50% of mutant calls; −, wild type.

b Allelic profile: *abcZ*, 59; *adk*, 39; *aroE*, 67; *fumC*, 156; *gdh*, 150; *pdhC*, 153; *pgm*, 133.

Analysis of the two highly AZT-resistant strains (MIC>256 mg/liter) confirmed the presence the A2059G mutation in the 23s rRNA gene as previously reported (2). Three and four copies of this mutant allele were inferred in the AZT-resistant ST1580 strains from 2008 and 2014, respectively, through assessment of the percentage reads supporting mutant allele calls (ca. 75% versus 100% supporting calls in the 2008 versus 2014 isolate relative to the NCCP11945 23s rRNA sequence). In addition, 355 variant loci differed between the 2008 and 2014 ST1580 isolates. Interestingly, the majority of these variants (314/355) occurred within a 7,715 bp region known to contain phase-variable antigenic factors including pilin (*opa*) and exopolyphosphatase (*ppx*) genes in NCCP11945 (NGK_0745-0755) (8). Thus, this putative antigen-switching event differentiated the temporally isolated highly AZT-resistant ST1580 strains, which also differed from each other at 41 other sites broadly distributed throughout the genome. Among five non-ST1580 strains exhibiting lower AZT resistance levels (MIC 50 = 8 mg/liter), an alternative AZT resistance mutation in the 23s rRNA (C2611T) was observed, whereas strains with neither the A2059G nor C2611T mutations were less resistant again (*n* = 8, MIC 50 = 1 mg/liter). An exception to this trend observed in a single strain (NGSJH13, MIC = 16 mg/liter) lacking any known resistance mutations in the 23s rRNA gene. This strain harbored a distinguishing loss of function mutation in *mtrR*, which could potentially account for its increased resistance, as functional loss of the MtrR regulator has been linked to AZT resistance in this species (9). These data provide a foundation for future surveillance of resistant *N. gonorrhoeae* in Ireland and internationally and highlights mechanisms of resistance and antigenic variability among AZT-resistant strains.

### Accession number(s).

This whole-genome shotgun project has been deposited at DDBJ/EMBL/GenBank under the accession numbers given in Table 1.
